# Factors influencing outcomes among patients with whiplash-associated disorder: A population-based study in Japan

**DOI:** 10.1371/journal.pone.0216857

**Published:** 2019-05-14

**Authors:** Kazuhiro Hayashi, Kenji Miki, Tatsunori Ikemoto, Takahiro Ushida, Masahiko Shibata

**Affiliations:** 1 Pain medicine and Research Information Center, Nonprofit Organization, Aichi, Japan; 2 Multidisciplinary Pain Center, Aichi Medical University, Aichi, Japan; 3 Department of Rehabilitation, Aichi Medical University Hospital, Aichi, Japan; 4 Department of Pain Medicine, Osaka University Graduate School of Medicine, Osaka, Japan; 5 Center for pain management, Hayaishi Hospital, Osaka, Japan; 6 Institute of Physical Fitness, Sports Medicine and Rehabilitation, Aichi Medical University, Aichi, Japan; Monash University, AUSTRALIA

## Abstract

**Introduction:**

Only a few, large population-based studies, have reported on whiplash-associated disorder (WAD). However, none of them have investigated the influence of crash severity on WAD outcome. In the present study, we aimed to determine whether crash severity predicts outcomes among patients with no-fault government insurance for acute WAD.

**Methods:**

We utilized data from a compulsory, no-fault government automobile liability insurance agency in Japan. Individuals involved in a car accident between April 2001 and June 2015 with residual disabilities reported at the end of the treatment between October 2014 and September 2015 were included. Crash severity was assessed based on property damage costs, size of the other vehicle (large car, medium car, small car, or two-wheeled vehicles), and collision types (rear-end collision, contact with vehicle moving in the same direction, or in the opposite direction). Outcomes included the time to claim closure and the number of treatment visits.

**Results:**

We analyzed data for a total of 52,251 individuals (28,571 male and 23,680 female) with a median age of 44 years (range: 2–95 years). The median time to claim closure was 220 days (range: 1–4,938 days), and the median number of treatment visits was 102 (range: 1–2,492). There was no significant association between outcomes and property damage costs or size of the other vehicle. Collision types exhibited no consistent association with outcomes. However, older age and affected body parts, in addition to the neck, were independent risk factors for delayed claim closure and a large number of visits, although, all odds ratios were low (often less than 2.0).

**Conclusions:**

There was no obvious association of outcomes with property damage costs, size of the other vehicle, or collision types in acute WAD patients. Further studies should investigate the influence of psychological factors, compensation systems, and cultural conditions.

## Introduction

Whiplash-associated disorder (WAD) encompasses the bony or soft-tissue injuries triggered by accidents involving rapid acceleration or deceleration, such as those associated with motor vehicle accidents [1]. WAD can manifest through a variety of symptoms, including neck pain or stiffness, headache, radicular symptoms, and cognitive impairment, suggesting a multifactorial etiology in patients with persistent symptoms [1]. The prevalence of WAD has recently increased, particularly within industrialized countries [2–4], although the exact rate varies according to geographical location [5–8].

A systematic review suggested that pain and disability symptoms decrease rapidly in the initial months after the accident but show little improvement after 3 months have elapsed [9]. Up to 50% of patients report pain and/or disability 12 months after their injuries [10–13], increasing individual, economic, and social burdens [14]. The factors influencing WAD outcomes remain to be fully elucidated. Another systematic review revealed that crash severity factors show limited prognostic value for recovery in patients with WAD [15]. Pain and disability in patients with WAD are associated with symptoms, insurance compensation, anxiety, depression, and catastrophizing and post-traumatic stress disorder, rather than injury-related physical or mechanical factors [16–19]. The authors further noted the inconsistency of reported prognostic factors for outcomes in previous studies, emphasizing the need for more rigorous evidence. A major limitation of earlier studies is that findings were based on cohort studies rather than population-based studies. Patients with WAD may consult an accident and emergency department, a general practitioner, a specialist, or a hospital. Hence, clinic-based studies have a potential selection bias, because the decision to seek a medical consultation depends not only on severity of the injury, but also on the psychological distress caused by pain [20]. In Japan, the patient has free access to health care insurance, especially out-patient clinics; therefore, a medical consultation is mainly associated with the patient’s decision. Population-based studies include patients regardless of the injury severity and kind of medical institution at which they sought treatment; therefore, they eliminate the impact of selection bias.

To date, only a few large, population-based studies on WAD have been reported in Canada [12,13,21] and United States [22–24]. However, they did not investigate the influence of crash severity on WAD outcome. Thus, population-based studies have investigated whether crash severity is associated with the severity of injury, but not the time required for claim closure or the number of treatment visits, in patients compensated under a no-fault government insurance agency, for acute WAD in Saskatchewan, Canada [12,13]. Similarly, the National Highway Traffic Safety Administration; an agency of the Executive Branch of the United States government part of the Department of Transportation, reports national survey data. The National Highway Traffic Safety Administration showed the statistics for injury in each body part. The National Highway Traffic Safety Administration in 1994 [22], 2000 [23], and 2015 [24] demonstrated that greater property damage tends to be associated with higher levels of injury, as determined by the Maximum Abbreviated Injury Scale scores. This tendency is also noted in the neck region, although this could include not only WAD but also severe injuries, such as fracture. However, population-based studies have not investigated associations with crash severity, property damage, time to claim closure, and the number of treatment visits.

In Japan, the following distinct systems for automobile insurance have been implemented: Compulsory and voluntary; they are independent of the universal healthcare system. Compulsory automobile liability insurance has been implemented since 1955 under the Automobile Liability Security Law [25]. The General Insurance Rating Organization of Japan, undertakes the necessary procedures for residual disability claims under the compulsory insurance systems, providing continuous insurance for 92.3% of the automobiles in Japan, regardless of the accident-risk level of each driver [26]. The other is a unique insurance service system initiated by the Japan Agricultural Cooperatives group, a national organization of farmers, which includes both, compulsory and voluntary insurance systems, in accordance with the Agricultural Cooperative Society Law. Compulsory no-fault automobile liability insurance covers bodily injury (but not property damage) associated with all types of automobile accidents in Japan, paying out roughly 794 billion yen (approximately 7.22 billion USD) per year. Compulsory insurance accounts for 68% of the overall compensation for automobile accident victims (1.17 trillion yen, approximately 10.6 billion USD). The voluntary insurance is purchased in addition to compulsory insurance, by more than 70% of the Japanese population [26]. Voluntary insurance is paid out when the compensation for damage exceeds the amount payable under compulsory insurance. Damage due to automobile accidents is assessed by the police based on a standard set of clearly-defined and uniformly-applied criteria.

Thus, in the present study, we aimed to determine whether crash severity could be used to predict time to claim closure or the number of treatment visits among patients with no-fault government insurance for acute WAD in Japan.

## Materials and methods

### Data source

To collect information regarding potential confounders and outcomes, the present study utilized data from a compulsory, no-fault government automobile liability insurance agency in Japan. Data were supplied by the General Insurance Rating Organization of Japan.

### Participants

Inclusion criteria were as follows: (1) involvement in a car accident with rear-end collision, contact with a vehicle moving in the same, or opposite direction; (2) primary diagnosis of WAD following the accident; (3) treatment in a medical institution covered by compulsory automobile liability insurance; and (4) involvement in a car accident between April 2001 and June 2015 (median: April 2014; IQR: December 2013–August 2014) with residual disability reported at the end of the treatment between October 2014 and September 2015. Victims of car accidents, wherein the claim had not been closed were excluded. Patients with fractures, dislocations, or spinal cord injuries were also excluded from the analyses. This study was approved by the Ethics Committee of Osaka University Graduate School of Medicine (No. 17136).

### Data collection

We collected demographic data, including patient age, sex, and presence or absence of affected body parts, other than neck. Crash severity was assessed based on the cost of property damage [22–24], size of the other vehicle, and collision type. The other vehicle was classified on the basis of size into the following four types: large car (total vehicle weight of over 11 tons), medium car (total vehicle weight of 11 tons or less; engine size over 660 cm^3^), small car (engine size of 660 cm^3^ or less), and two-wheeled vehicles. Collision types were classified as follows: rear-end collision, contact with a vehicle moving in the same direction, or contact with one moving in the opposite direction. The latter, could be estimated to cause a serious injury.

We recorded the date of the first medical visit following injury, as well as primary outcomes including the time to claim closure and the number of treatment visits. Time to claim closure was regarded as the number of days between the date of injury and the date corresponding to the closure of the insurance claim, which has been verified as a valid marker of health recovery [12,13,27]. The number of treatment visits was recorded because compensation for treatment is based on this measure in Japan [28].

### Statistical analysis

The normality of the distribution for each measure was evaluated using the Shapiro-Wilk test for continuous variables. The time to claim closure and the number of treatment visits were not normally distributed. Continuous variables are represented as medians and interquartile ranges (IQR), while categorical variables are represented as the number and percentage of patients.

Patients were categorized into the following two groups according to time to claim closure, based on the findings of previous population-based studies [12,13,21]: a “delayed claim closure group”, among whom treatment spanned greater than 12 months, and a “normal claim closure group”, among whom treatment spanned ≤12 months. Patients were also classified into the following two groups based on the number of treatment visits, in accordance with a previous WAD study: a “large number of visits group” (upper 25%) and a “normal number of visits group” [29]. All collected variables were included in the multivariable logistic regression analysis. We calculated odds ratios and 95% confidence intervals (CIs) for all variables in the groups, via multivariable logistic regression analysis, to estimate prognostic factors for each treatment outcome. The data were analyzed using IBM SPSS Statistics Version 25.0 (IBM Corp., Armonk, NY, USA). A P value of < 0.05 was considered statistically significant.

We also performed a *post hoc* power analysis for each analysis using G*Power software (v 3.0.10; Franz Faul, Kiel University, Kiel, Germany).

## Results

**Fig 1:**
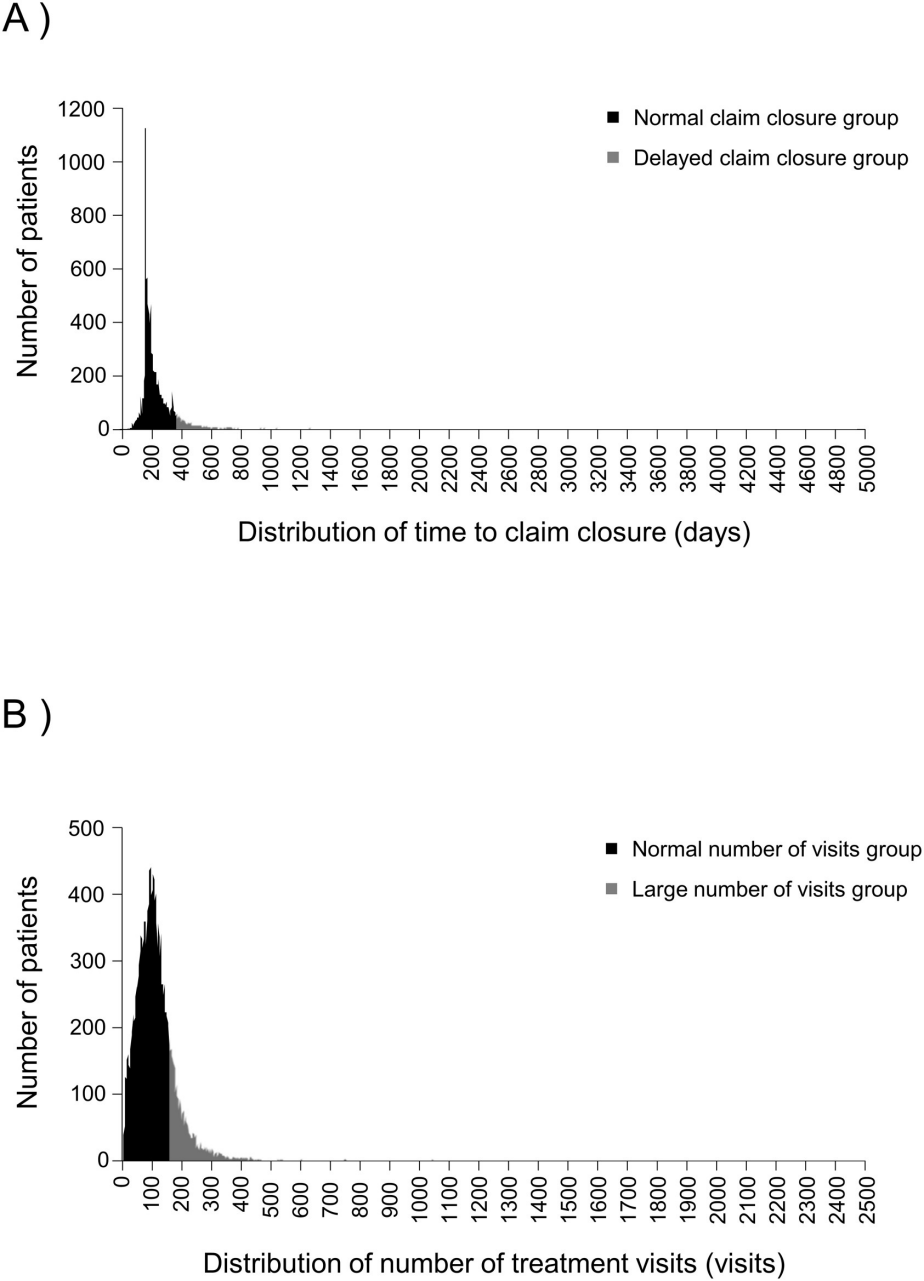
(A) Distribution of time to claim closure. (B) Distribution of number of treatment visits.

We analyzed data for a total of 52,251 individuals (28,571 male and 23,680 female) with a median age of 44 years (range: 2–95 years; IQR: 36–55 years). Patient characteristics are presented in Table 1. The median time to claim closure was 220 days (range: 1–4,938 days; IQR: 187–288 days) (Fig 1A), whereas the median number of treatment visits was 102 (range: 1–2,492, IQR: 68–140) (Fig 1B). The median cost of property damage was 230,000 yen (approximately 2,000 USD) (range: 0–40,000,000 yen; IQR: 0–500,000 yen). A total of 1,910 (4%), 35,690 (68%), 13,903 (27%), and 748 (1%) patients were involved in accidents involving large cars, medium cars, small cars, and two-wheeled vehicles, respectively. A total of 32,233 (62%), 3,095 (6%), and 16,923 (32%) patients were involved in rear-end collision, contact with a vehicle moving in the same direction, or contact with one moving in the opposite direction, respectively. *Post hoc* power analysis revealed that each analysis exhibited sufficient statistical power (99%).

**Table 1:** Patients’ characteristics (n = 52,251).

Female gender, n (%)	23,680 (45%)
Age (years)	44 [36–55]
19 ≤, n (%)	700 (1%)
20–29, n (%)	5,555 (11%)
30–39, n (%)	11,779 (23%)
40–49, n (%)	15,466 (30%)
50–59, n (%)	9,506 (18%)
60–69, n (%)	6,317 (12%)
≥ 70, n (%)	2,928 (6%)
Affected body parts in addition to the neck	
with head, n (%)	8,234 (16%)
with trunk, n (%)	35,230 (67%)
with limb, n (%)	25,379 (49%)
Property damage costs (yen)	230,000 [0–500,000]
Size of the other vehicle	
Large car, n (%)	1,910 (4%)
Medium car, n (%)	35,690 (68%)
Small car, n (%)	13,903 (27%)
Two wheels, n (%)	748 (1%)
Collision types	
rear-ended, n (%)	32,233 (62%)
contact with vehicle moving in the same direction, n (%)	3,095 (6%)
contact with vehicle moving in the opposite direction, n (%)	16,923 (32%)
Time to first visit (days)	0 [0–1]
0 day, n (%)	32,348 (62%)
1 day, n (%)	11,401 (22%)
2 days, n (%)	3,988 (8%)
more than 3 days, n (%)	4,514 (9%)
Number of treatment visits (visits)	102 [68–140]
Time to claim closure (days)	220 [187–288]

Data from continuous variables are shown in medians and interquartile ranges [IQR]. Data from categorical variables are shown in number and (%) of patients.

Table 2 shows the differences between groups based on time to claim closure (delayed recovery group (n = 475) and normal recovery group (n = 51,776)). Univariate analyses revealed significant differences in gender, age, affected body part, in addition to the neck, and collision types between the groups. In the multivariate analysis, female gender, older age, and affected body part, in addition to the neck, were identified as independent risk factors for delayed recovery, although all odds ratios were low (often less than 2.0). The cost of property damage and size of the other vehicle exhibited no significant associations with the time to claim closure in either the univariate or multivariate analyses. Collision types exhibited no consistent association with time to claim closure.

**Table 2:** Difference between groups based on time to claim closure.

	Univariate				Multivariate	
	Delayed claim closure group (n = 475)	Normal claim closure group (n = 51,776)	Odds ratio (95%CI)	p-Values	Odds ratio (95%CI)	p-Value
Female gender, n (%)	242 (51%)	23,438 (45%)	**1.256 (1.048–1.505)**	0.014*	**1.200 (1.001–1.439)**	0.049*
Age (years)	48 [41–60]	44 [36–55]	**1.022 (1.016–1.029)**	<0.001*		
19 ≤, n (%)	4 (1%)	696 (1%)	0.623 (0.232–1.672)	0.348		0.166
20–29, n (%)	15 (3%)	5,540 (11%)	**0.272 (0.163–0.455)**	<0.001*	**0.245 (0.146–0.411)**	<0.001*
30–39, n (%)	81 (17%)	11,698 (23%)	**0.704 (0.554–0.895)**	0.004*	**0.653 (0.511–0.834)**	0.001*
40–49, n (%)	167 (35%)	15,299 (30%)	**1.293 (1.070–1.562)**	0.008*		0.371
50–59, n (%)	87 (18%)	9,419 (18%)	1.008 (0.798–1.274)	0.944		0.256
60–69, n (%)	68 (14%)	6,249 (12%)	1.217 (0.940–1.576)	0.136		0.473
≥ 70, n (%)	53 (11%)	2,875 (6%)	**2.136 (1.601–2.849**	<0.001*	**1.726 (1.287–2.316)**	<0.001*
Affected body part in addition to the neck						
with head, n (%)	128 (27%)	8,106 (16%)	**1.987 (1.620–2.437)**	<0.001*	**1.834 (1.493–2.254)**	<0.001*
with trunk, n (%)	373 (79%)	34,857 (67%)	**1.775 (1.425–2.211)**	<0.001*	**1.735 (1.390–2.165)**	0.001*
with limb, n (%)	290 (61%)	25,089 (48%)	**1.667 (1.385–2.007)**	<0.001*	**1.517 (1.258–1.830)**	<0.001*
Property damage costs (yen)	88,500 [0–413,679]	230,112 [0–500,000]	0.998 (0.996–1.000)	0.063		0.094
Size of the other vehicle						
Large car, n (%)	13 (3%)	1,897 (4%)	0.740 (0.426–1.286)	0.286		0.203
Medium car, n (%)	333 (70%)	35,357 (68%)	1.089 (0.894–1.327)	0.397		0.351
Small car, n (%)	120 (25%)	13,783 (27%)	0.932 (0.757–1.147)	0.505		0.544
Two wheels, n (%)	9 (2%)	739 (1%)	1.334 (0.687–2.590)	0.395		0.526
Collision types						
Rear-ended, n (%)	265 (56%)	31,968 (62%)	**0.782 (0.652–0.938)**	0.008*		0.239
Contact with vehicle moving in the same direction, n (%)	32 (7%)	3,063 (6%)	1.149 (0.801–1.648)	0.451		0.696
Contact with vehicle moving in the opposite direction, n (%)	178 (37%)	16,745 (32%)	**1.254 (1.040–1.511)**	0.018*		0.317
Time to first visit (days)	0 [0–1]	0 [0–1]	**0.870 (0.802–0.943)**	0.001*		0.137
0 day, n (%)	352 (74%)	31,996 (62%)				
1 day, n (%)	74 (16%)	11,327 (22%)				
2 days, n (%)	25 (5%)	3,963 (8%)				
more than 3 days, n (%)	24 (5%)	4,490 (9%)				

Data from continuous variables are shown in medians and interquartile ranges [IQR]. Data from categorical variables are shown in number and (%) of patients.

*Significant difference between delayed claim closure group and normal claim closure group (p < 0.05).

Table 3 shows the differences between groups based on the number of treatment visits (large number of visits (n = 12,975) and normal number of visits (n = 39,276)). Older age, affected body part, in addition to the neck, and earlier time to first visit were identified as independent risk factors for a large number of visits in both the univariate and multivariate analyses, although all odds ratios were low (often less than 1.6). The cost of property damage, size of the other vehicle, and collision type exhibited no significant association with the number of treatment visits in either the univariate or multivariate analyses.

**Table 3:** Difference between groups based on the number of treatment visits.

	Univariate				Multivariate	
	Large number of visits group (n = 12,975)	Normal number of visits group (n = 39,276)	Odds ratio (95%CI)	p-Value	Odds ratio (95%CI)	p-Value
Female gender, n (%)	5,865 (45%)	17,815 (45%)	0.994 (0.955–1.034)	0.757		0.164
Age (years)	46 [38–57]	44 [35–54]	**1.014(1.012–1.015)**	<0.001*		
19 ≤, n (%)	108 (1%)	592 (2%)	**0.548 (0.446–0.674)**	<0.001*	**0.510 (0.414–0.628)**	<0.001*
20–29, n (%)	976 (8%)	4,579 (12%)	**0.616 (0.573–0.662)**	<0.001*	**0.577 (0.535–0.622)**	<0.001*
30–39, n (%)	2,637 (20%)	9,142 (23%)	**0.841 (0.801–0.883)**	<0.001*	**0.799 (0.758–0.841)**	<0.001*
40–49, n (%)	4,006 (31%)	11,460 (29%)	**1.084 (1.038–1.132)**	<0.001*		0.451
50–59, n (%)	2,474 (19%)	7,032 (18%)	**1.080 (1.027–1.137)**	0.003*		0.451
60–69, n (%)	1,848 (14%)	4,469 (11%)	**1.294 (1.220–1.371)**	<0.001*	**1.219 (1.146–1.297)**	<0.001*
≥ 70, n (%)	926 (7%)	2,002 (5%)	**1.431 (1.320–1.551)**	<0.001*	**1.332 (1.225–1.449)**	<0.001*
Affected body part in addition to the neck						
with head, n (%)	2,429 (19%)	5,805 (15%)	**1.328 (1.261–1.399)**	<0.001*	**1.251 (1.186–1.320)**	<0.001*
with trunk, n (%)	9,671 (75%)	25,559 (65%)	**1.571 (1.502–1.643)**	<0.001*	**1.553 (1.484–1.625)**	<0.001*
with limb, n (%)	6,932 (53%)	18,447 (47%)	**1.295 (1.245–1.348)**	<0.001*	**1.220 (1.171–1.271)**	<0.001*
Property damage costs (yen)	220,000 [0–500,000]	232,670 [0–500,000]	1.000 (1.000–1.000)	0.577		0.161
Size of the other vehicle						
Large car, n (%)	496 (4%)	1,414 (1%)	1.064 (0.959–1.181)	0.242		0.898
Medium car, n (%)	8,815 (68%)	26,875 (68%)	0.978 (0.937–1.020)	0.301		0.449
Small car, n (%)	3,465 (27%)	10,438 (27%)	1.007 (0.962–1.053)	0.773		0.722
Two wheels, n (%)	199 (2%)	549 (1%)	1.099 (0.933–1.294)	0.259		0.148
Collision types						
rear-ended, n (%)	7,964 (61%)	24,269 (62%)	0.983 (0.943–1.024)	0.403		0.214
contact with vehicle moving in the same direction, n (%)	776 (6%)	2,319 (6%)	1.014 (0.932–1.102)	0.749		0.576
contact with vehicle moving in the opposite direction, n (%)	4,235 (33%)	12,688 (32%)	1.015 (0.973–1.059)	0.480		0.118
Time to first visit (days)	0 [0–1]	0 [0–1]	**0.913 (0.900–0.927)**	<0.001*	**0.927 (0.914–0.941)**	<0.001*
0 days, n (%)	8,680 (67%)	23,668 (60%)				
1 day, n (%)	2,641 (20%)	8760 (22%)				
2 days, n (%)	828 (6%)	3,160 (8%)				
more than 3 days, n (%)	826 (6%)	3,688 (9%)				

Data from continuous variables are shown in medians and interquartile ranges [IQR]. Data from categorical variables are shown in number and (%) of patients.

*Significant difference between large number of visits group and normal number of visits group (p < 0.05).

## Discussion

The present population-based study investigated the impact of crash severity on outcomes in patients with no-fault government insurance for acute WAD. Our study suggested that there is no obvious association of property damage, size of the other vehicle, and collision type with time to claim closure or the number of treatment visits in patients with WAD. However, our analysis indicated that older age and affected body part, in addition to the neck, are slightly associated with a delayed claim closure and a large number of visits.

Previous systematic reviews have concluded that there is limited association [15] or no association [16] with crash severity factors and patient recovery. The evidence for such associations is inconsistent [17]. Other studies have indicated that the severity of injury is associated with collision characteristics, the characteristics of other vehicles involved [30,31], and higher unit costs of property damage [22–24]. Meanwhile, the results of the present study suggested that there is no obvious association between crash severity and outcomes. WAD often occurs as a result of rear-end vehicle collisions at speeds of less than 14 mph [32]. Also, perturbations due to low-velocity rear-end motor vehicle accidents are similar to those encountered during daily living [33].

A systematic review of acute WAD has reported controversial evidence regarding the effect of age on outcomes [16]. Older patients often experience pain prior to the accident, which may influence acute WAD outcomes [16]. Our findings demonstrated that older age is an independent risk factor for both delayed claim closure and a large number of treatment visits among patients with acute WAD, although the odds ratios for both outcomes were small. Future studies should investigate this association while accounting for initial pain intensity, anxiety, depression, and catastrophizing and post-traumatic stress disorder [16–19].

Among subjects with WAD alone, 25% recover within 1 week of their crash, while 1.9% do not recover even 1 year after their crash [34]. On the other hand, among subjects with other injuries in addition to WAD, 19% recover within 1 week, while 4.1% do not recover even 1 year after their crash [34]. Similarly, the present study showed that another body part affected in addition to the neck, was a significant independent risk factor for a delayed claim closure and a large number of visits.

Previous studies have also reported that early healthcare utilization is associated with continued pain and disability among patients with acute WAD [16]. Such studies have also demonstrated that greater health care utilization is associated with delayed recovery from acute WAD [12,13,21,35]. In accordance with these findings, the present study revealed that earlier time to first visit is an independent risk factor for a large number of treatment visits, regardless of crash severity. Frequent testing and visits to doctors have been found to provide little reassurance and increase feelings of worry and anxiety among patients [36]. Reliance on clinical care may have a negative effect on recovery by promoting the use of passive coping strategies [6,37].

The prevalence of WAD continues to increase, particularly within most of Europe, North America, Australia, and Asia [2–4], ranging from 16 to 200 per 100,000 individuals based on the geographical location [5–8]. Cultural conditions and symptom expectations are thought to influence the prevalence of WAD [10]. For example, prevalence rates are low in Lithuania, Germany, and Greece, due to people in those countries having low symptom expectations for the chronic outcome of WAD [7,8,38–40]. In Northern Sweden, WAD-related insurance claims have exhibited a rapid decrease during the past 7–8 years [41]. Previous studies have highlighted that WADs have been less frequently discussed in the Swedish media during the past 7–8 years, which may have reduced awareness regarding such injuries [41]. Meanwhile, in Saskatchewan, Canada, the type of insurance system exerts a profound effect on the frequency and duration of whiplash claims, and previous studies have reported that claimants recover faster when compensation for pain and suffering is unavailable [2]. Similarly, the legislative change to reduce compensation for disability for WAD in Australia has shown a significant improvement in health status, as assessed in relation to disability, pain and physical functioning [42]. In the present study, we analyzed data from 52,251 patients with residual disability reported at the end of the treatment due to WAD in a single year, which suggests a prevalence of 43 per 100,000 individuals, although this may be an underestimation [43]. In Japan, the number of patients with neck injuries due to WAD from January to December 2014 was 403,846 [44]. The cost of treatment following automobile accidents is covered by insurance companies rather than patients in Japan. The compensation is based on the number of medical visits, in addition to residual disability and lost earnings [28]. This may explain increases in the number of medical visits and time to claim closure, regardless of injury-related physical or mechanical factors. Future studies should thus examine the influence of the compensation system and cultural conditions on the prevalence and outcomes of acute WAD.

The present study possesses several notable limitations. First, the medical expenses of car accident casualties in Japan may include not only those covered by public car insurance but also those covered by public and private medical insurance. Although time to claim closure is based on the end of the treatment, it does not necessarily indicate time to recovery. Some patients may finalize their claim despite incomplete recovery [45]. Patients may also continue to experience pain and receive treatment even after closure of the compulsory, no-fault government automobile liability insurance claim. In addition, the patient may not claim for the accident at all, if the disability is deemed as not being caused in a car accident. Second, we did not assess crash severity based on the actual force of impact, relying instead on the cost of property damage, size of the other vehicle, and collision types. Third, the outcomes included not only WAD but also injuries other than those involving the neck, even though patients received a primary diagnosis of WAD. Fourth, we did not investigate the therapeutic approaches utilized (e.g., medication, rehabilitation, education, multidisciplinary treatment, etc.), or the association between compensation and symptoms. Indeed, previous studies have reported that acute WAD outcomes are associated with compensation, post-injury pain, disability, and psychological factors [16]. Finally, only 92.3% of automobiles in Japan are covered by the General Insurance Rating Organization. The other, a unique insurance service system initiated by the Japan Agricultural Cooperatives group, mostly provides insurance for farmers in rural areas. Moreover, the present study excluded those victims of car accidents, whose claim had not been closed. The present study could have underestimated WAD patients’ and victim’s emotional request for a harsher penalty.

## Conclusions

The present population-based study demonstrated no obvious association of outcomes with property damage costs, size of the other vehicle, or collision types in patients with acute WAD. However, older age and affected body part, in addition to the neck, were identified as independent risk factors for delayed claim closure and a large number of visits. Further studies should investigate the influence of psychological factors, compensation systems, and cultural conditions among patients with acute WAD.
